# Cardiac disease mechanobiology: advances using hiPSC-CMs

**DOI:** 10.3389/fcvm.2025.1642931

**Published:** 2025-09-29

**Authors:** Georgina Aluoch Stephanie, Alison Schroer Vander Roest

**Affiliations:** ^1^ Department of Chemical Engineering, University of Michigan, Ann Arbor, MI, United States; ^2^ Department of Biomedical Engineering, University of Michigan, Ann Arbor, MI, United States

**Keywords:** hiPSC-CMs, mechanobiology, disease modeling, engineered platforms, cardiomyopathies

## Abstract

Cardiovascular diseases (CVDs) remain the leading cause of mortality worldwide. Despite advances in diagnosis and treatment, there is a critical need for sophisticated models that accurately reflect human cardiovascular pathophysiology. This mini review explores recent advancements in cardiac microenvironment engineering for modeling cardiac mechanobiology and investigating genetic and acquired cardiac diseases. Cardiac function relies heavily on mechanical cues, with integrin- and cadherin-based adhesion complexes mediating mechanosensitive signaling that drives disease progression. However, studying these processes in humans remains challenging. Although animal models have been indispensable, they often fail to recapitulate human-specific cardiac features. Human-induced pluripotent stem cells (hiPSCs) have been transformative, enabling patient-specific modeling and the identification of disease-specific phenotypes that are challenging to replicate in traditional animal models. Despite their promise, hiPSC-CMs are constrained by their immature phenotype and heterogeneity, which limits their efficacy in modeling adult cardiac physiology. Emerging *in vitro* systems, particularly those engineered using biomaterials such as hydrogels, address these limitations by mimicking the mechanical and biochemical environment of native cardiac tissue. We discuss the potential and challenges of these hiPSC-derived cardiomyocytes (hiPSC-CMs) in modeling cardiac mechanotransduction, focusing on the interplay between mechanical stress and cellular maturation, mechanics, and signaling. By integrating advanced biomaterials and genome editing technologies, these *in vitro* platforms hold the potential to revolutionize cardiac research, offering the prospect of more precise interventions and improved patient outcomes.

## Introduction

Cardiovascular diseases are characterized by altered mechanical function and remain the leading cause of mortality globally despite progress in diagnosis and treatment (1). The field of molecular genetics has identified key mutations and pathways involved in disease onset and progression, improving diagnosis and informing therapies (2). Additionally, tissue engineering has revolutionized research on cardiac mechanobiology­­­­—the process by which cardiac cells sense and respond to mechanical stimuli *in vitro* (3). By leveraging advances in material science and cell culture techniques, *in vitro* cardiac tissue models that mimic the native heart environment can elucidate cardiac physiology and pathology.

**Figure 1:**
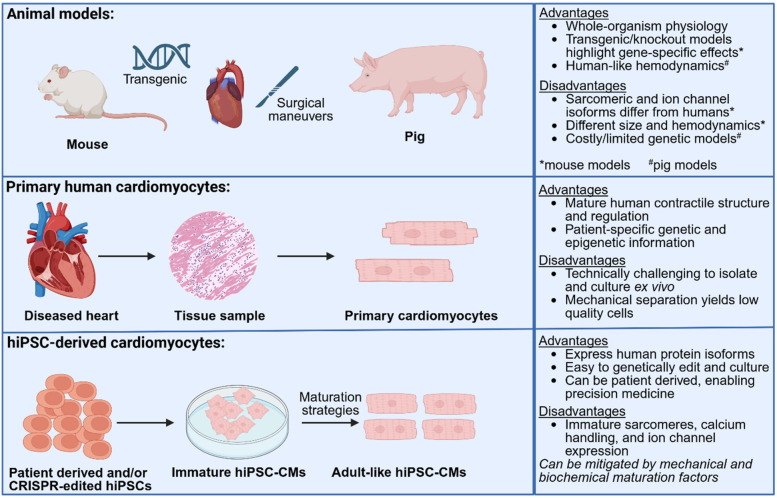
Schematic overview of three commonly used platforms: animal models, primary human cardiomyocytes, and hiPSC-CMs. Large (e.g., pig) and small (e.g., mouse) animal models enable whole-organism studies and genetic manipulation, but are limited by species-specific differences and difficulty in modeling human disease heterogeneity. Primary cardiomyocytes from patient tissue retain native structure and genotype but are technically challenging to isolate and culture. hiPSC-CMs offer scalable, patient-specific platforms for modeling cardiac disease and drug response, though they exhibit fetal-like features without appropriate maturation. Each model presents unique advantages and limitations that inform their use in cardiovascular research. Created in BioRender. UIUC, J. (2025) https://BioRender.com/a66j7s6.

Animal models have been pivotal in advancing our understanding of cardiovascular diseases but have significant practical and biological limitations. Surgical maneuvers including transaortic constriction or shunts have been used in both large and small animal models to induce pressure or volume overload, respectively. These procedures trigger dysfunction and cardiac remodeling, highlighting cardiac sensitivity to changes in mechanics in disease (4). Mouse models are commonly used due to their short reproductive cycles, low cost and ease of genetic manipulation. However, species-specific differences in cardiac physiology and genetic composition limit their translational relevance (5–8). For example, two common transgenic models of inherited hypertrophic cardiomyopathy (HCM)—R403Q-MyHC and R92W-TnT—show marked differences in gene expression, mitochondrial function, and redox balance, not only from each other but also from human myectomy tissue, with minimal overlap in dysregulated genes or pathways between species (8). These findings highlight that genotype alone is insufficient for predicting phenotype or therapeutic responses in cardiac disease modeling. Large animal models such as pigs match the physiology, heart size, immune system and cardiac anatomy of humans, but are limited by the difficulty of introducing disease-causing mutations as well as high rates of sudden cardiac death due to tacharrhythmias (9–11). While primary human cardiomyocytes retain relevant, native structural and electrophysiological properties, their limited availability, poor viability in culture and lack of scalability pose significant challenges for disease modeling. These limitations underscore the need for complementary models that balance physiological relevance with experimental accessibility (Figure 1).

Human induced pluripotent stem cells (hiPSCs) offer a scalable, patient-specific platform that enables mechanistic insights into cardiac pathophysiology (12, 13). Additionally, genome-editing approaches such as CRISPR-Cas9 can be used to repair disease genotypes in patient-derived hiPSCs or introduce relevant mutations to study their pathophysiology (14–17). Improvements in the efficiency of hiPSC production processes, from the harvesting of somatic cells for hiPSC generation, to the differentiation of cardiomyocytes and other cardiac cell types, have driven advancements in drug discovery, disease modeling, and tissue engineering.

Furthermore, engineered 2D and 3D *in vitro* models that mimic the composition and organization of the cardiac extracellular matrix (ECM), have revolutionized cardiac disease modeling, drug screening technologies, and regenerative medicne (18–21). Hydrogels have become increasingly popular as platforms for modeling cardiac diseases *in vitro*, addressing the limitations of traditional tissue culture plastic, which fail to replicate the complex physiological and dynamic environment of cardiac tissue. Their potential for biofunctionalization, tissue-like elasticity and tunable mechanical properties make hydrogels a suitable platform for modeling cardiac disease, thus facilitating the study of cardiomyocyte function, cell-matrix interactions, cardiac mechanobiology and thus disease progression *in vitro* (22–24). Leveraging these biomaterial platforms allows for the understanding of cardiomyocyte mechanosensing in both healthy and diseased myocardium.

## Myocardial mechanobiology: a double-edged sword

The heart undergoes continuous contraction and relaxation cycles and must be able to respond and adapt to varying mechanical stress and hemodynamic load throughout an animal's lifespan. This is enabled by complex remodeling processes that are mediated by both cardiomyocytes and non-myocytes. Cells respond to mechanical cues transduced into biochemical signaling cascades to promote remodeling under physiological and pathological conditions, a process known as mechanotransduction or mechanosensing (25, 26).

### Mechanobiology in cardiac development and physiological remodeling

The heart experiences significant changes in load and demand during pre- and post-natal development, increasing in size via hypertrophy and maturation of cardiomyocytes. As the heart grows to meet increased circulatory needs, cardiomyocytes experience continuous mechanical stress from the heart's beating and the surrounding ECM. These biomechanical cues are sensed through mechanotransduction pathways, which coordinate sarcomeric growth and alignment, contributing to the functional organization of the myocardium (27). Under normal conditions, mechanotransduction is a compensatory mechanism that maintains homeostasis as the cell adapts to an altered mechanical environment.

The cytoskeleton plays a crucial role in the mechanotransduction process, linking the cellular and contractile machinery to force-sensing complexes that interface with the ECM. Costameres align with z disks in cardiac muscle and function as attachment sites for transmission of lateral forces to the ECM. They comprise a network of mechanosensitive proteins, including integrins, that coordinate contraction of the heart muscle and are crucial for mechanosensing. Integrins are a family of cell-surface receptors composed of α and β subunits with extracellular domains that bind an ECM ligand while their cytoplasmic domains engage cytoskeletal and force-sensitive proteins like talin, paxillin, and vinculin (28). These domains also recruit intracellular signaling mediators including focal adhesion kinase, IQGAP1, and melusin, initiating cascades such as the Raf-MEK-ERK pathway, which culminates in ERK phosphorylation and cardiomyocyte hypertrophy (29, 30). Beyond signal transduction, integrins contribute to adhesion stability and cytoskeletal organization, influencing cell shape, size, and morphology. The dominant integrins in cardiomyocytes—α1β1, α5β1, and α7β1—bind to collagen, fibronectin, and laminin, respectively (31). Notably, integrin expression is developmentally regulated; the α5 subunit is prevalent in fetal and neonatal cardiomyocytes but is later replaced by α7 during maturation. Integrin *α*5 has been shown to be critical for maturation of hiPSC-CMs cultured in 3D hydrogels. Its knockdown impaired epithelial-to-mesenchymal transition, reduced expression of key mesodermal markers (T Brachyury, TBX6), and diminished cardiomyocyte differentiation and contractility. These findings highlight the importance of integrin-linked signaling pathways in shaping hiPSC-CM development and maturation (32).

HiPSC-CMs replated onto collagen I at later stages of differentiation have also been shown to exhibit a shift in integrin isoform expression—particularly upregulating α1 and β1 subunits—accompanied by activation of FAK and ERK signaling pathways. This integrin-mediated mechanotransduction cascade drives structural and electrophysiological maturation, including increased expression of adult myosin isoforms and improved calcium handling (33). In parallel, focal adhesion-associated kinases, such as FAK and members of the MAPK family (ERK, JNK, p38), have also been shown to be key modulators of cardiomyocyte specification from pluripotent stem cells, linking external mechanical signals to lineage commitment via phosphorylation of transcriptional and epigenetic regulators. These kinases integrate ECM and growth factor cues to guide mesodermal induction, cardiac progenitor formation, and sarcomeric gene activation, positioning them as essential nodes in both mechanobiological signaling and cardiogenesis (34). Moreover, integrin-linked kinases (ILKs) are critical for cytoskeletal remodeling in reprogramming. iPSC kinome-wide functional analysis suggests a critical role of integrin in the cytoskeletal remodelling process. Specifically, integrin-linked kinases within the integrin-linked kinase (ILK) network play a crucial role in cellular reprogramming through cytoskeletal remodeling (35). These findings highlight the critical role of extracellular matrix composition and the temporal dynamics of cell-matrix interactions in modulating integrin-mediated signaling cascades in development and disease modeling.

Additionally, cell adhesion proteins such as N-cadherin, have been shown to play a critical role in maintaining the structural integrity and function of cardiomyocytes as part of adherens junctions at intercalated discs. N-cadherin binds and stabilizes β-catenin, which is a crucial component of the adherens junction involved in the WNT signaling pathway (36).Standardized hiPSC-CM differentiation protocols primarily target the WNT signaling pathway to direct mesodermal and cardiac lineage specification (37–39).

### Mechanobiology in cardiomyopathy and pathological remodeling

Under abnormal conditions, mechanotransduction can drive maladaptive responses and pathological remodeling (40–43). Recent studies using primary human cardiomyocytes from patients with heart failure have shown that cytoskeletal forces are relayed to the nucleus via desmin and microtubule networks, and that disruption of this architecture can lead to chromatin reorganization, altered gene expression and impaired contractile function (44). Additionally, recent studies leveraging hiPSC-CMs have highlighted the centrality of cytoskeletal components such as filamin C (FLNC), in maintaining these mechanical linkages. FLNC truncation variants disrupt cytoskeletal stiffness, impair cell–ECM adhesion, and induce arrhythmic beating profiles in a gene-dosage-dependent manner. Atomic force microscopy analysis revealed compromised mechanical integrity at both the membrane and cytoplasmic levels, underscoring the importance of costameric and cytoskeletal integrity for proper mechanosignaling (45). Changes in ECM composition and mechanical integrity due to cardiac injuries or genetic cardiomyopathies often result in changes in integrin expression patterns. For instance, integrin expression has been shown to revert to fetal isoforms in murine models, with upregulation of α5, α7, and β1D (46, 47). Additionally, loss of integrin-linked kinase expression in cardiomyocytes has been linked with arrhythmogenic cardiomyopathy in mice (48).

Interestingly, increased β1-integrin expression at the sarcolemma of N-cadherin-deficient murine cardiomyocytes suggests enhanced cell-ECM interactions as an adaptive mechanism for providing additional structural stability in the absence of intact adherens junctions (49). Targeted N-Cadherin deletion in CMs has also been shown to impede cardiac regeneration in neonatal mice, leading to excessive scarring following ischemic injury (50). However, the mechanisms through which these changes drive remodeling at the microscale level are challenging to resolve *in vivo*. Furthermore, the pathophysiology of many cardiomyopathies manifests differently in animal models. For instance, volume overload caused by extended endurance training can exacerbate the chance of arrhythmia in human patients with ACM-associated mutations, including mutations in the cell-cell junction protein plakophilin 2 (PKP2). However, prolonged volume depletion protected mice with ACM-prone PKP2 mutations from developing arrhythmias, even with prolonged exercise (51). An hiPSC-CM model of arrhythmogenesis showed exaggerated lipogenesis and apoptosis in mutant PKP2 hiPSC-CMs. Furthermore, hiPSC-CMs with a homozygous PKP2 mutation also had calcium-handling deficits potentially associated with arrythmia, a phenotype not observed in ACM mouse models (52). Additionally, mechanosensitive ion channels that are crucial for excitation-contraction recoupling have also been shown to be implicated in pathological remodeling. Recent studies of HCM hiPSC-CMs cultured on stiff polyacrylamide gels have identified hypermetabolic mitochondrial remodeling in HCM due to disrupted calcium handling between the L-type calcium channel and mitochondria (40). These findings further emphasize the need for *in vitro* platforms that incorporate human-derived cells and allow for spatial and molecular resolution of mechanotransductional remodeling during cardiac pathophysiology.

## Engineered *in vitro* systems for cardiac mechanobiology: challenges and opportunities

The application of hiPSC-CMs to *in vitro* model systems is constrained by their immature, embryonic-like phenotype and intrapopulation heterogeneity, limiting their ability to model adult cardiac physiology (53). For example, hiPSC-CMs have decreased, hierarchical organization of myofibrils, which is crucial in the myocardium, where normal function is highly coupled to tissue organization and mechanics. hiPSC-CM immaturity is further characterized by differences in electrophysiological properties, ion channel expression, and metabolic profiles. This immaturity has been attributed to the lack of *in vivo* environmental cues that the cells experience during development. For example, hiPSC-derived cardiomyocytes cultured on traditional tissue plastic as monolayers often lack the anisotropic alignment of myofibrils, which is crucial for mechanical transduction and electrical activity (54, 55). The native myocardium has a Young's modulus of 10–18 kPa under physiological conditions, whereas a diseased myocardium can stiffen beyond 50 kPa (56). The Young's modulus of most traditional tissue culture plastics is approximately 1 GPa, about 5–6 orders of magnitude stiffer than even the diseased myocardium. Studies have also shown that both ECM composition and density are crucial in modulating cellular mechanics as well as downstream signaling cascades that promote homeostasis (57, 58). This therefore establishes a need for physiologically relevant materials that allow for resolution of cardiac mechanobiological remodeling under both physiological and pathological conditions. Biomimetic materials have been explored for better replication of the cardiac microenvironment. Key biomaterial features such as stiffness, topography, and viscoelasticity have been shown to critically influence cardiomyocyte alignment, maturation, and force generation. Both synthetic hydrogels such as polyacrylamide gels, and natural hydrogels such as collagen and hyaluronic acid have been explored as alternatives to tissue plastic for *in vitro* cell culture (59–61).

In addition to bulk material properties, spatial cues have proven to be critical for hiPSC-CM culture. Microfabrication techniques such as microcontact printing (µCP) have been employed to engineer spatially patterned substrates that provide alignment cues and promote myofibril alignment and cardiomyocyte maturation *in vitro*. Single-cell micropatterning techniques afford precise control over cell shape, polarity, and spatial confinement. Rod-shaped micropatterns, designed to mimic the elongated morphology of native cardiomyocytes not only enhance structural organization but also significantly improve electrophysiological maturation, including increased sodium current density, faster action potential upstroke velocity, and improved calcium handling (62). Traction force microscopy analysis of single-cell hiPSC-CMs has also demonstrated that enhanced myofibrillar organization leads to greater sarcomeric activity, improving contractile output as measured by microbead displacement during contraction-relaxation cycles (63). Taken together, these findings show that substrates with physiological stiffness significantly improve contractile activity of patterned hiPSC-CMs, calcium transients, electrophysiology, mitochondrial organization, and transverse-tubule formation and facilitate improved disease modeling *in vitro*.

The tunability of hydrogel platforms has also enabled mechanistic insights into pathological remodeling linked to disease-associated changes in tissue stiffness. For example, increasing polyacrylamide substrate stiffness exacerbates Duchenne muscular dystrophy (DMD) hiPSC-CM dysfunction by impairing calcium handling, reducing contractile force, and inducing contraction-dependent telomere shortening. The resulting telomere attrition activates a p53-mediated DNA damage response, linking mechanical stress to progressive cardiomyopathy and identifying telomere maintenance as a potential therapeutic target (64).

While single-cell microcontact printing has been shown to achieve highly consistent single hiPSC-CM geometry, variable contractile function has been reported, potentially due to a lack of connectivity to neighboring cardiomyocytes. Patterns that allow for multi-cellular contact have been used to promote connectivity and enhance mechanical and electrical coupling. For example, innovations in micropatterning, such as the integration of ECM geometry with N-cadherin “end caps” on hydrogels, have been shown to reshape cardiomyocyte morphology with minimal impact on contractile function, suggesting that cell–cell adhesion cues may fine-tune architectural features without substantially altering baseline mechanical output (21). Additionally, developmentally inspired fibronectin micropatterns derived from embryonic heart ECM have been shown to guide cardiomyocyte alignment in a density- and cell-type–dependent manner, mediated in part by N-cadherin–based cell–cell interactions, thus emphasizing the need for spatially and temporally controlled microenvironments that recapitulate both cell–cell and cell–ECM interactions (65).

Importantly, the spatiotemporal dynamics of cell–cell junction assembly have also been shown to be critical for normal hiPSC-CM development. Adherens junctions and desmosomes assemble progressively in response to mechanical and organizational cues, and their maturation is dependent on proper spatial alignment. Disruption of desmosomal proteins in hiPSC-CMs derived from patients with PKP2 mutations impairs junctional integrity, force transmission, and contractile coordination. Notably, these defects can be partially rescued by modulating the mechanical microenvironment to promote cytoskeletal alignment, focal adhesion organization, and enhanced cell-cell contact, reinforcing the concept that junction assembly is tightly regulated by mechanotransduction and cellular architecture (15). Thus, biophysical context, such as cellular alignment and force transmission, modulates the severity of these phenotypes, offering insight into mechanotransduction-dependent pathogenesis.

Further, culture substrates that promote robust, mechanically organized cardiac muscle tissue formation yet still allow for contractile analysis in 2D have been reported. Micropatterning of elastic polydimethylsiloxane (PDMS) to anchor thin, purified cardiac muscle strips in 2D arrays showed that myofibrillar alignment and physiological contractions in an optimized media environment drive improved development of contractile function, calcium handling, and electrophysiology (66).

**Figure 2:**
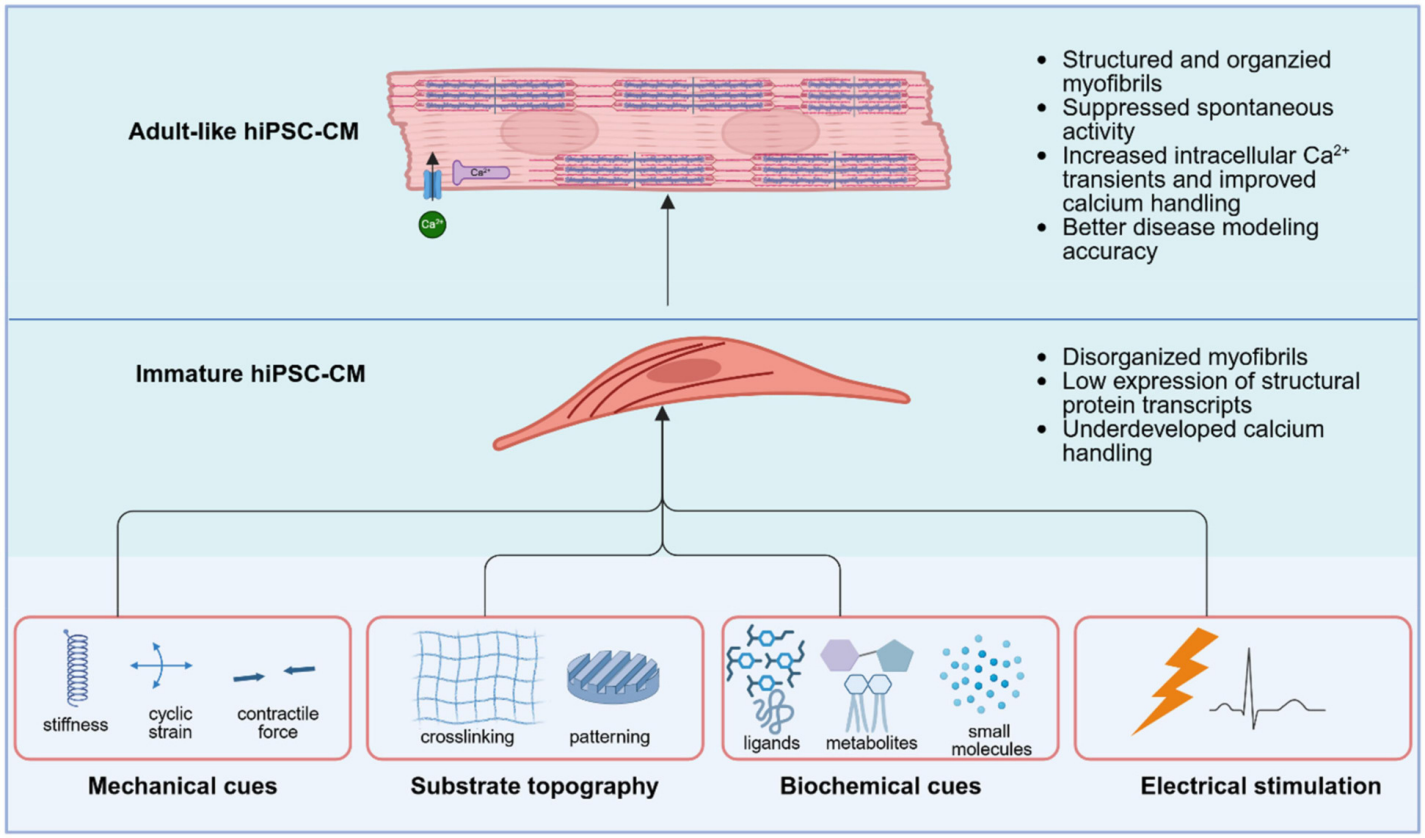
Strategies to promote functional maturation of hiPSC-derived cardiomyocytes. Immature hiPSC-CMs exhibit disorganized myofibrils, low expression of structural protein transcripts, and underdeveloped calcium handling, which limit their utility for accurate disease modeling. Multiple bioengineering strategies have been developed to drive their maturation toward an adult-like phenotype, characterized by organized sarcomeres, suppressed spontaneous activity, and enhanced intracellular calcium transients. These strategies include mechanical cues (e.g., substrate stiffness, cyclic strain, and contractile loading), substrate topography (e.g., ECM-crosslinked micropatterning), biochemical modulation and electrical stimulation. Integration of these cues promotes structural, electrophysiological, and metabolic maturation of hiPSC-CMs, thereby improving their fidelity as human cardiac disease models. Created in BioRender. UIUC, J. (2025) https://BioRender.com/a66j7s6.

Additionally, recent work has also shown that applying cyclic mechanical stretch to hiPSC-CMs cultured on collagen I-coated PDMS membranes enhances sarcomeric organization, contractility, and gene expression through nuclear mechanotransduction, reinforcing the importance of mechanical cues in guiding maturation via chromatin remodeling and transcriptional activation (67). Electrical stimulation has also emerged as a crucial tool in promoting the structural and functional maturation of hiPSC-CMs. For example, it has been observed that electrical stimulation applied during differentiation promotes hiPSC-CMs into a specialized conduction system phenotype, marked by increased expression of connexin 40. Additionally, electrically stimulated hiPSC-CMs exhibited faster action potential depolarization, longer intracellular Ca2+ transients, and slower action potential duration at 90% of repolarization (68). In addition to mechanical and electrical stimulation, chemically defined maturation strategies have been developed to emulate the adult cardiac metabolic environment. For example, protocols incorporating reduced glucose, increased fatty acid concentrations (e.g., palmitate, oleate), and galactose have been shown to shift hiPSC-CM metabolism from glycolysis to oxidative phosphorylation, leading to increased mitochondrial content, enhanced membrane potential, and upregulated fatty acid oxidation enzymes and expression of maturation markers (69–71). In conjunction with bioengineering approaches, these hiPSC maturation strategies have improved the electrophysiological, structural, and metabolic fidelity of hiPSC-CMs, advancing their utility in cardiac disease modeling (Figure 2).

Extending beyond 2D systems, optimized 3D ECM environments, defined by specific combinations of matrix composition, stiffness, and timing of cell–ECM engagement, significantly enhance murine cardiomyocyte differentiation from embryonic stem cells. Using a statistical modeling framework, fibronectin-based matrices with intermediate stiffness and early exposure timing have been shown to be optimal for promoting cardiac gene expression and sarcomeric organization, reinforcing the importance of ECM mechanobiology in guiding lineage commitment and maturation (72).

Additionally, engineered heart tissues (EHTs), have revolutionized the field of cardiac mechanobiology, have been crucial for modeling the mechanical behavior of the myocardium (73–75). Cell-dense hydrogels, typically a mixture of fibrin and collagen, are cast between two stretching posts. These posts provide mechanical stretching, thus facilitating the modeling of preload and afterload dynamics. EHTs have also been integrated to provide a heart-on-a-chip strategy, wherein engineered, anisotropic ventricular myocardium with muscular thin film are multiplexed to provide real-time, parallel physiological and pharmacological assessment across an ensemble of tissues (76). For example, it has been shown that dynamic loading of wild-type EHTs improves contractile force, enhanced alignment, conduction velocity and contractility. Interestingly, it has also been shown that dynamic loading is necessary to recapitulate ACM due to mutations in the desmoplakin gene (73). Collectively, these platforms have advanced our ability to model biomechanical cues and disease-specific phenotypes *in vitro*, offering a scalable framework for probing the mechanistic underpinnings of cardiac function and dysfunction with high spatial and physiological fidelity.

Beyond disease modeling, hiPSCs’ regenerative and pluripotent capacity are also being heavily explored for regenerative therapies. Initial preclinical studies explored various stem cell sources for cardiac repair in animal models. These studies demonstrated that intramyocardial injection of mouse iPSCs following coronary ligation improves cardiac function and attenuates remodeling in immunocompetent mice (77). However, other studies observed teratoma formation in immunodeficient mice, raising concerns about potential risks associated with transplantation of undifferentiated iPSCs (78). To address this, differentiation protocols have been refined to derive committed cardiovascular lineages such as cardiomyocytes, endothelial cells, and smooth muscle cells prior to transplantation, reducing the risk of tumor formation. However, a few challenges remain, such as poor engraftment and limited cell retention at the infarcted site due to rapid clearance. Strategies have shifted towards using tissue-engineered constructs, where hiPSC-derived cardiomyocytes are encapsulated within bioactive hydrogels that mimic the native myocardial ECM. For instance, the development of a multicellular cardiac patch consisting of hiPSC-derived cardiomyocytes, smooth muscle cells, endothelial cells and cardiac fibroblasts, significantly enhanced myocardial repair in a porcine model of myocardial infarction (79). Improved left ventricular ejection fraction, vascular density, enhanced engraftment of mature human cardiomyocytes, and electromechanical integration with host tissue were observed, without the induction of arrhythmias. These findings underscore the promise of hiPSC-derived tissue constructs as next-generation therapeutic platforms for cardiac regeneration.

## Conclusions and future perspectives

The use of iPSC-derived cardiomyocytes (CMs) to model cardiac pathophysiology and understand cardiac mechanobiology has ushered in a new era of potential insights into the intricacies of heart disease. These models have allowed researchers to overcome the limitations posed by traditional animal models and primary human cardiac cells, providing a more accurate representation of human-specific cardiac conditions, genetic influences, and patient-specific phenotypes. Despite the ongoing limitations of hiPSCs, advancements in biomaterials, especially hydrogels, alongside genome editing technologies like CRISPR-Cas9, have highlighted their potential in mimicking the physiological and pathological conditions of the human heart. This has allowed the study of mechanobiological signaling pathways and their role in regulating cardiomyocyte maturation, contractility, and disease phenotypes *in vitro*. Future research could focus on refining biochemical maturation strategies of hiPSC-CMs as well as enhancing the robustness microengineered cardiac tissues to replicate the dynamics of the native cardiac environment more faithfully.

## References

[1] Joseph P Leong D McKee M Anand SS Schwalm J-D Teo K Reducing the global burden of cardiovascular disease, part 1. Circ Res. (2017) 121:677–94. 10.1161/CIRCRESAHA.117.308903 28860318

[2] Lam CK Wu JC. Disease modelling and drug discovery for hypertrophic cardiomyopathy using pluripotent stem cells: how far have we come? Eur Heart J. (2018) 39:3893–5. 10.1093/eurheartj/ehy388 30010924 PMC6234848

[3] Zhao Y Feric NT Thavandiran N Nunes SS Radisic M. The role of tissue engineering and biomaterials in cardiac regenerative medicine. Can J Cardiol. (2014) 30(11):1307–22. 10.1016/j.cjca.2014.08.027 25442432 PMC4254531

[4] Pilz PM Ward JE Chang W-T Kiss A Bateh E Jha A Large and small animal models of heart failure with reduced ejection fraction. Circ Res. (2022) 130:1888–905. 10.1161/CIRCRESAHA.122.320246 35679365

[5] Santini L Palandri C Nediani C Cerbai E Coppini R. Modelling genetic diseases for drug development: hypertrophic cardiomyopathy. Pharmacol Res. (2020) 160:105176. 10.1016/j.phrs.2020.105176 32871247

[6] Gerull B Brodehl A. Genetic animal models for arrhythmogenic cardiomyopathy. Front Physiol. (2020) 11:624. 10.3389/fphys.2020.00624 32670084 PMC7327121

[7] van den Dolder FW Dinani R Warnaar VAJ Vučković S Passadouro AS Nassar AA Experimental models of hypertrophic cardiomyopathy: a systematic review. JACC Basic Transl Sci. (2025) 10:511–46. 10.1016/j.jacbts.2024.10.017 40306862 PMC12134605

[8] Vakrou S Liu Y Zhu L Greenland GV Simsek B Hebl VB Differences in molecular phenotype in mouse and human hypertrophic cardiomyopathy. Sci Rep. (2021) 11:13163. 10.1038/s41598-021-89451-6 34162896 PMC8222321

[9] Spannbauer A Traxler D Zlabinger K Gugerell A Winkler J Mester-Tonczar J Large animal models of heart failure with reduced ejection fraction (HFrEF). Front Cardiovasc Med. (2019) 6:117. 10.3389/fcvm.2019.00117 31475161 PMC6702665

[10] Low GKK Azahar A Samson E Rane P. Systematic review of swine models for ventricular fibrillation induction in evaluating cardiopulmonary resuscitation methods. Cardiol Plus. (2024) 9:91. 10.1097/CP9.0000000000000087

[11] Bauer J Vlcek J Pauly V Hesse N Xia R Mo L Biomarker periodic repolarization dynamics indicates enhanced risk for arrhythmias and sudden cardiac death in myocardial infarction in pigs. J Am Heart Assoc. (2024) 13:e032405. 10.1161/JAHA.123.032405 38639363 PMC11179938

[12] Karakikes I Ameen M Termglinchan V Wu JC. Human induced pluripotent stem cell–derived cardiomyocytes. Circ Res. (2015) 117:80–8. 10.1161/CIRCRESAHA.117.305365 26089365 PMC4546707

[13] Karagiannis P Takahashi K Saito M Yoshida Y Okita K Watanabe A Induced pluripotent stem cells and their use in human models of disease and development. Physiol Rev. (2019) 99:79–114. 10.1152/physrev.00039.2017 30328784

[14] Joshi J Albers C Smole N Guo S Smith SA. Human induced pluripotent stem cell-derived cardiomyocytes (iPSC-CMs) for modeling cardiac arrhythmias: strengths, challenges and potential solutions. Front Physiol. (2024) 15:1475152. 10.3389/fphys.2024.1475152 39328831 PMC11424716

[15] Kim SL Trembley MA Lee KY Choi S MacQueen LA Zimmerman JF Spatiotemporal cell junction assembly in human iPSC-CM models of arrhythmogenic cardiomyopathy. Stem Cell Rep. (2023) 18:1811–26. 10.1016/j.stemcr.2023.07.005 PMC10545490 37595583

[16] Vander Roest AS Liu C Morck MM Kooiker KB Jung G Song D Hypertrophic cardiomyopathy *β*-cardiac myosin mutation (P710R) leads to hypercontractility by disrupting super relaxed state. Proc Natl Acad Sci U S A. (2021) 118:e2025030118. 10.1073/pnas.2025030118 34117120 PMC8214707

[17] Ewoldt JK Wang MC McLellan MA Cloonan PE Chopra A Gorham J Hypertrophic cardiomyopathy–associated mutations drive stromal activation via EGFR-mediated paracrine signaling. Sci Adv. (2024) 10:eadi6927. 10.1126/sciadv.adi6927 39413182 PMC11482324

[18] Ganguli A Mostafa A Saavedra C Kim Y Le P Faramarzi V Three-dimensional microscale hanging drop arrays with geometric control for drug screening and live tissue imaging. Sci Adv. (2021) 7:eabc1323. 10.1126/sciadv.abc1323 33893093 PMC8064630

[19] Andrysiak K Stępniewski J Dulak J. Human-induced pluripotent stem cell-derived cardiomyocytes, 3D cardiac structures, and heart-on-a-chip as tools for drug research. Pflüg Arch Eur J Physiol. (2021) 473:1061–85. 10.1007/s00424-021-02536-z PMC8245367 33629131

[20] Brazhkina O Park JH Park H-J Bheri S Maxwell JT Hollister SJ Designing a 3D printing based auxetic cardiac patch with hiPSC-CMs for heart repair. J Cardiovasc Dev Dis. (2021) 8(12):172. 10.3390/jcdd8120172 34940527 PMC8706296

[21] Lane KV Dow LP Castillo EA Boros R Feinstein SD Pardon G Cell architecture and dynamics of human induced pluripotent stem cell-derived cardiomyocytes (hiPSC-CMs) on hydrogels with spatially patterned laminin and N-cadherin. ACS Appl Mater Interfaces. (2025) 17:174–86. 10.1021/acsami.4c11934 39680735 PMC11783353

[22] Dong Y Hong M Dai R Wu H Zhu P. Engineered bioactive nanoparticles incorporated biofunctionalized ECM/silk proteins based cardiac patches combined with MSCs for the repair of myocardial infarction: *in vitro* and *in vivo* evaluations. Sci Total Environ. (2020) 707:135976. 10.1016/j.scitotenv.2019.135976 31865091

[23] Tallawi M Rosellini E Barbani N Cascone MG Rai R Saint-Pierre G Strategies for the chemical and biological functionalization of scaffolds for cardiac tissue engineering: a review. J R Soc Interface. (2015) 12:20150254. 10.1098/rsif.2015.0254 26109634 PMC4528590

[24] Cheng Y He C Xiao C Ding J Cui H Zhuang X Versatile biofunctionalization of polypeptide-based thermosensitive hydrogels via click chemistry. Biomacromolecules. (2013) 14:468–75. 10.1021/bm3017059 23311471

[25] Lyon RC Zanella F Omens JH Sheikh F. Mechanotransduction in cardiac hypertrophy and failure. Circ Res. (2015) 116:1462–76. 10.1161/CIRCRESAHA.116.304937 25858069 PMC4394185

[26] Barrick SK Greenberg MJ. Cardiac myosin contraction and mechanotransduction in health and disease. J Biol Chem. (2021) 297:101297. 10.1016/j.jbc.2021.101297 34634306 PMC8559575

[27] Wang C Ramahdita G Genin G Huebsch N Ma Z. Dynamic mechanobiology of cardiac cells and tissues: current status and future perspective. Biophysics Rev. (2023) 4(1):011314. 10.1063/5.0141269 PMC10062054 37008887

[28] Valencik ML Zhang D Punske B Hu P McDonald JA Litwin SE. Integrin activation in the heart. Circ Res (2006) 99:1403–10. 10.1161/01.RES.0000252291.88540.ac 17095723

[29] Gallo S Vitacolonna A Bonzano A Comoglio P Crepaldi T. ERK: a key player in the pathophysiology of cardiac hypertrophy. Int. J. Mol. Sci. (2019) 20:2164. 10.3390/ijms20092164 31052420 PMC6539093

[30] Nakhaei-Rad S Haghighi F Bazgir F Dahlmann J Busley AV Buchholzer M Molecular and cellular evidence for the impact of a hypertrophic cardiomyopathy-associated RAF1 variant on the structure and function of contractile machinery in bioartificial cardiac tissues. Commun Biol. (2023) 6:657. 10.1038/s42003-023-05013-8 37344639 PMC10284840

[31] Israeli-Rosenberg S Manso AM Okada H Ross RS. Integrins and integrin-associated proteins in the cardiac myocyte. Circ. Res. (2014) 114:572–86. 10.1161/CIRCRESAHA.114.301275 24481847 PMC3975046

[32] Neiman G Scarafía MA La Greca A Santín Velazque NL Garate X Waisman A Integrin alpha-5 subunit is critical for the early stages of human pluripotent stem cell cardiac differentiation. Sci Rep. (2019) 9:18077. 10.1038/s41598-019-54352-2 31792288 PMC6889169

[33] Barreto-Gamarra C Domenech M. Integrin stimulation by collagen I at the progenitor stage accelerates maturation of human iPSC-derived cardiomyocytes. J Mol Cell Cardiol. (2025) 201:70–86. 10.1016/j.yjmcc.2025.02.009 40023481

[34] Robert S Flowers M Ogle BM. Kinases of the focal adhesion complex contribute to cardiomyocyte specification. Int J Mol Sci. (2021) 22:10430. 10.3390/ijms221910430 34638793 PMC8508671

[35] Sakurai K Talukdar I Patil VS Dang J Li Z Chang K-Y Kinome-wide functional analysis highlights the role of cytoskeletal remodeling in somatic cell reprogramming. Cell Stem Cell. (2014) 14:523–34. 10.1016/j.stem.2014.03.001 24702998 PMC4071169

[36] Manring HR Dorn LE Ex-Willey A Accornero F Ackermann MA. At the heart of inter- and intracellular signaling: the intercalated disc. Biophys Rev. (2018) 10:961–71. 10.1007/s12551-018-0430-7 29876873 PMC6082301

[37] Burridge PW Keller G Gold JD Wu JC. Production of *de novo* cardiomyocytes: human pluripotent stem cell differentiation and direct reprogramming. Cell Stem Cell. (2012) 10:16–28. 10.1016/j.stem.2011.12.013 22226352 PMC3255078

[38] Chen VC Ye J Shukla P Hua G Chen D Lin Z Development of a scalable suspension culture for cardiac differentiation from human pluripotent stem cells. Stem Cell Res. (2015) 15:365–75. 10.1016/j.scr.2015.08.002 26318718 PMC4600677

[39] Lyra-Leite DM Gutiérrez-Gutiérrez Ó Wang M Zhou Y Cyganek L Burridge PW. A review of protocols for human iPSC culture, cardiac differentiation, subtype-specification, maturation, and direct reprogramming. STAR Protoc. (2022) 3:101560. 36035804 10.1016/j.xpro.2022.101560 PMC9405110

[40] Viola HM Richworth C Solomon T Chin IL Szappanos HC Sundararaj S A maladaptive feedback mechanism between the extracellular matrix and cytoskeleton contributes to hypertrophic cardiomyopathy pathophysiology. Commun Biol. (2023) 6:1–13. 10.1038/s42003-022-04278-9 36596888 PMC9810744

[41] Eden M Kilian L Frank D Frey N. Cardiac mechanoperception and mechanotransduction: mechanisms of stretch sensing in cardiomyocytes and implications for cardiomyopathy. In: Hecker M Duncker DJ, editors. Cardiac Mechanobiology in Physiology and Disease. Cham: Springer International Publishing (2023). p. 1–35. 10.1007/978-3-031-23965-6_1

[42] Garoffolo G Pesce M. Mechanotransduction in the cardiovascular system: from developmental origins to homeostasis and pathology. Cells. (2019) 8:1607. 10.3390/cells8121607 31835742 PMC6953076

[43] Pesce M Duda GN Forte G Girao H Raya A Roca-Cusachs P Cardiac fibroblasts and mechanosensation in heart development, health and disease. Nat Rev Cardiol. (2023) 20:309–24. 10.1038/s41569-022-00799-2 36376437

[44] Chen CY Caporizzo MA Bedi K Vite A Bogush AI Robison P Suppression of detyrosinated microtubules improves cardiomyocyte function in human heart failure. Nat Med. (2018) 24:1225–33. 10.1038/s41591-018-0046-2 29892068 PMC6195768

[45] Lazzarino M Zanetti M Chen SN Gao S Peña B Lam CK Defective biomechanics and pharmacological rescue of human cardiomyocytes with filamin C truncations. Int J Mol Sci. (2024) 25:2942. 10.3390/ijms25052942 38474188 PMC10932268

[46] Nawata J Ohno I Isoyama S Suzuki J Miura S Ikeda J Differential expression of *α*1, *α*3 and *α*5 integrin subunits in acute and chronic stages of myocardial infarction in rats. Cardiovasc Res. (1999) 43:371–81. 10.1016/S0008-6363(99)00117-0 10536667

[47] Babbitt CJ Shai S-Y Harpf AE Pham CG Ross RS. Modulation of integrins and integrin signaling molecules in the pressure-loaded murine ventricle. Histochem. Cell Biol. (2002) 118:431–9. 10.1007/s00418-002-0476-1 12483308

[48] Quang KL Maguy A Qi X-Y Naud P Xiong F Tadevosyan A Loss of cardiomyocyte integrin-linked kinase produces an arrhythmogenic cardiomyopathy in mice. Circ Arrhythm Electrophysiol. (2015) 8:921–32. 10.1161/CIRCEP.115.001668 26071395

[49] Kostetskii I Li J Xiong Y Zhou R Ferrari VA Patel VV Induced deletion of the N-cadherin gene in the heart leads to dissolution of the intercalated disc structure. Circ Res. (2005) 96:346–54. 10.1161/01.RES.0000156274.72390.2c 15662031

[50] Tsai Y-W Tseng Y-S Wu Y-S Song W-L You M-Y Hsu Y-C N-Cadherin promotes cardiac regeneration by potentiating pro-mitotic *β*-catenin signaling in cardiomyocytes. Nat Commun. (2025) 16:896. 10.1038/s41467-025-56216-y 39837836 PMC11751462

[51] Fabritz L Hoogendijk MG Scicluna BP van Amersfoorth SCM Fortmueller L Wolf S Load-Reducing therapy prevents development of arrhythmogenic right ventricular cardiomyopathy in plakoglobin-deficient mice. J Am Coll Cardiol. (2011) 57:740–50. 10.1016/j.jacc.2010.09.046 21292134

[52] Kim C Wong J Wen J Wang S Wang C Spiering S Studying arrhythmogenic right ventricular dysplasia with patient-specific iPSCs. Nature. (2013) 494:105–10. 10.1038/nature11799 23354045 PMC3753229

[53] Koivumäki JT Naumenko N Tuomainen T Takalo J Oksanen M Puttonen KA Structural immaturity of human iPSC-derived cardiomyocytes: in silico investigation of effects on function and disease modeling. Front Physiol. (2018) 9:80. 10.3389/fphys.2018.00080 PMC5808345 29467678

[54] Shi H Wang C Gao BZ Henderson JH Ma Z. Cooperation between myofibril growth and costamere maturation in human cardiomyocytes. Front Bioeng Biotechnol. (2022) 10:1049523. 10.3389/fbioe.2022.1049523 36394013 PMC9663467

[55] Napiwocki BN Lang D Stempien A Zhang J Vaidyanathan R Makielski JC Aligned human cardiac syncytium for *in vitro* analysis of electrical, structural, and mechanical readouts. Biotechnol Bioeng. (2021) 118:442–52. 10.1002/bit.27582 32990953 PMC8214444

[56] Montero P Flandes-Iparraguirre M Musquiz S Pérez Araluce M Plano D Sanmartín C Cells, materials, and fabrication processes for cardiac tissue engineering. Front Bioeng Biotechnol. (2020) 8:955. 10.3389/fbioe.2020.00955 32850768 PMC7431658

[57] Trappmann B Baker BM Polacheck WJ Choi CK Burdick JA Chen CS. Matrix degradability controls multicellularity of 3D cell migration. Nat Commun. (2017) 8:371. 10.1038/s41467-017-00418-6 28851858 PMC5575316

[58] Gao L Kupfer ME Jung JP Yang L Zhang P Da Sie Y Myocardial tissue engineering with cells derived from human-induced pluripotent stem cells and a native-like, high-resolution, 3-dimensionally printed scaffold. Circ Res. (2017) 120:1318–25. 10.1161/CIRCRESAHA.116.310277 28069694 PMC5392171

[59] Zhang Q Wang P Fang X Lin F Fang J Xiong C. Collagen gel contraction assays: from modelling wound healing to quantifying cellular interactions with three-dimensional extracellular matrices. Eur J Cell Biol. (2022) 101:151253. 10.1016/j.ejcb.2022.151253 35785635

[60] Pryse KM Nekouzadeh A Genin GM Elson EL Zahalak GI. Incremental mechanics of collagen gels: new experiments and a new viscoelastic model. Ann Biomed Eng. (2003) 31:1287–96. 10.1114/1.1615571 14649502

[61] Yasmeen N Karpinska A Kalecki J Kutner W Kwapiszewska K Sharma PS. Electrochemically synthesized polyacrylamide gel and core–shell nanoparticles for 3D cell culture formation. ACS Appl Mater Interfaces. (2022) 14:32836–44. 10.1021/acsami.2c04904 35848208 PMC9335524

[62] Al Sayed ZR Jouve C Seguret M Ruiz-Velasco A Pereira C Trégouët D-A Rod-shaped micropatterning enhances the electrophysiological maturation of cardiomyocytes derived from human induced pluripotent stem cells. Stem Cell Rep. (2024) 19:1417–31. 10.1016/j.stemcr.2024.08.005 PMC11561463 39303707

[63] Ribeiro AJS Ang Y-S Fu J-D Rivas RN Mohamed TMA Higgs GC Contractility of single cardiomyocytes differentiated from pluripotent stem cells depends on physiological shape and substrate stiffness. Proc Natl Acad Sci U S A. (2015) 112:12705–10. 10.1073/pnas.1508073112 26417073 PMC4611612

[64] Chang ACY Pardon G Chang ACH Wu H Ong S-G Eguchi A Increased tissue stiffness triggers contractile dysfunction and telomere shortening in dystrophic cardiomyocytes. Stem Cell Rep. (2021) 16:2169–81. 10.1016/j.stemcr.2021.04.018 PMC8452491 34019816

[65] Batalov I Jallerat Q Kim S Bliley J Feinberg AW. Engineering aligned human cardiac muscle using developmentally inspired fibronectin micropatterns. Sci Rep. (2021) 11:11502. 10.1038/s41598-021-87550-y 34075068 PMC8169656

[66] DePalma SJ Davidson CD Stis AE Helms AS Baker BM. Microenvironmental determinants of organized iPSC-cardiomyocyte tissues on synthetic fibrous matrices. Biomater. Sci. (2021) 9:93–107. 10.1039/D0BM01247E 33325920 PMC7971708

[67] Song M Jang Y Kim S-J Park Y. Cyclic stretching induces maturation of human-induced pluripotent stem cell-derived cardiomyocytes through nuclear-mechanotransduction. Tissue Eng Regen Med. (2022) 19:781–92. 10.1007/s13770-021-00427-z 35258794 PMC9294081

[68] Crestani T Steichen C Neri E Rodrigues M Fonseca-Alaniz MH Ormrod B Electrical stimulation applied during differentiation drives the hiPSC-CMs towards a mature cardiac conduction-like cells. Biochem Biophys Res Commun. (2020) 533:376–82. 10.1016/j.bbrc.2020.09.021 32962862

[69] Hsueh Y-C Pratt RE Dzau VJ Hodgkinson CP. Novel method of differentiating human induced pluripotent stem cells to mature cardiomyocytes via Sfrp2. Sci Rep. (2023) 13:3920. 10.1038/s41598-023-31144-3 36894665 PMC9998650

[70] Feyen M McKeithan DA N WL Bruyneel AA Spiering S Hörmann L Metabolic maturation Media improve physiological function of human iPSC-derived cardiomyocytes. Cell Rep. (2020) 32:107925. 10.1016/j.celrep.2020.107925 32697997 PMC7437654

[71] Vučković S Dinani R Nollet EE Kuster DWD Buikema JW Houtkooper RH Characterization of cardiac metabolism in iPSC-derived cardiomyocytes: lessons from maturation and disease modeling. Stem Cell Res Ther. (2022) 13:332. 10.1186/s13287-022-03021-9 35870954 PMC9308297

[72] Jung JP Hu D Domian IJ Ogle BM. An integrated statistical model for enhanced murine cardiomyocyte differentiation via optimized engagement of 3D extracellular matrices. Sci Rep. (2015) 5:18705. 10.1038/srep18705 26687770 PMC4685314

[73] Bliley JM Vermeer MCSC Duffy RM Batalov I Kramer D Tashman JW Dynamic loading of human engineered heart tissue enhances contractile function and drives a desmosome-linked disease phenotype. Sci Transl Med. (2021) 13:eabd1817. 10.1126/scitranslmed.abd1817 34290054

[74] Leonard A Bertero A Powers JD Beussman KM Bhandari S Regnier M Afterload promotes maturation of human induced pluripotent stem cell derived cardiomyocytes in engineered heart tissues. J Mol Cell Cardiol. (2018) 118:147–58. 10.1016/j.yjmcc.2018.03.016 29604261 PMC5940558

[75] Cofiño-Fabres C Boonen T Rivera-Arbeláez JM Rijpkema M Blauw L Rensen PCN Micro-Engineered heart tissues on-chip with heterotypic cell composition display self-organization and improved cardiac function. Adv Healthc Mater. (2024) 13:2303664. 10.1002/adhm.202303664 38471185

[76] Grosberg A Alford PW McCain ML Parker KK. Ensembles of engineered cardiac tissues for physiological and pharmacological study: heart on a chip. Lab. Chip. (2011) 11:4165–73. 10.1039/c1lc20557a 22072288 PMC4038963

[77] Nelson TJ Martinez-Fernandez A Yamada S Perez-Terzic C Ikeda Y Terzic A. Repair of acute myocardial infarction by human stemness factors induced pluripotent stem cells. Circulation. (2009) 120:408–16. 10.1161/CIRCULATIONAHA.109.865154 19620500 PMC2768575

[78] Ahmed RP Ashraf M Buccini S Shujia J Haider HK. Cardiac tumorgenic potential of induced pluripotent stem cells in an immunocompetent host with myocardial infarction. Regen Med. (2011) 6(2):171–8. 10.2217/rme.10.103 21391851 PMC3110348

[79] Lou X Tang Y Ye L Pretorius D Fast VG Kahn-Krell AM Cardiac muscle patches containing four types of cardiac cells derived from human pluripotent stem cells improve recovery from cardiac injury in mice. Cardiovasc Res. (2023) 119:1062–76. 10.1093/cvr/cvad004 36647784 PMC10153642

